# The Dynamics of Networks of Identical Theta Neurons

**DOI:** 10.1186/s13408-018-0059-7

**Published:** 2018-02-05

**Authors:** Carlo R. Laing

**Affiliations:** Institute of Natural and Mathematical Sciences, Massey University, Auckland, New Zealand

**Keywords:** Theta neurons, Watanabe/Strogatz ansatz, Bifurcation

## Abstract

We consider finite and infinite all-to-all coupled networks of identical theta neurons. Two types of synaptic interactions are investigated: instantaneous and delayed (via first-order synaptic processing). Extensive use is made of the Watanabe/Strogatz (WS) ansatz for reducing the dimension of networks of identical sinusoidally-coupled oscillators. As well as the degeneracy associated with the constants of motion of the WS ansatz, we also find continuous families of solutions for instantaneously coupled neurons, resulting from the reversibility of the reduced model and the form of the synaptic input. We also investigate a number of similar related models. We conclude that the dynamics of networks of all-to-all coupled identical neurons can be surprisingly complicated.

## Introduction

Due to their analytical intractability, many investigations of large networks of model neurons involve extensive numerical simulation [1–4]. Without any detailed knowledge of the connectivity between neurons, one might assume the simplest form of connectivity: all-to-all. One might also (again, for simplicity) assume that the neurons are identical. Typically such highly-symmetric networks have as attractors either perfect synchrony or more general synchrony, in which neurons follow the same periodic orbit, but with phase shifts between them. Other possible attractors are cluster states in which some subsets of neurons are perfectly synchronised, or partial synchrony in which individual oscillators show quasiperiodic behaviour while the network as a whole is periodic [5, 6]. The possible dynamics of such networks can be very complicated due to the symmetry of the system [7, 8].

Perfect synchrony corresponds to a form of dimension reduction, since the network is effectively replaced by a single self-coupled neuron. However, the stability of this state depends on the linearisation of the full dynamics around it. In this paper we use another form of dimension reduction, the Watanabe/Strogatz (WS) ansatz [9, 10], applicable to all-to-all coupled networks of identical phase oscillators. For the ansatz to be applicable, the velocity field of an oscillator has to contain only the first harmonics of the phase variable. The theta neuron [11, 12] is such a model oscillator.

The derivation of a network of coupled phase oscillators from a general weakly-coupled network of oscillators is well known [7, 13, 14]. Unlike those derivations, the equation for a theta neuron, involving just one phase variable, is the normal form of a saddle-node-on-a-circle (SNIC) bifurcation, and will thus describe all neurons undergoing this bifurcation, at least in some neighbourhood in a parameter space of the bifurcation. Such a bifurcation leads to the neuron being of Type I [15, 16], i.e. capable of firing at arbitrarily low frequencies.

A number of previous authors have used the WS ansatz in order to study the dynamics of networks of identical all-to-all coupled phase oscillators, but the underlying oscillator models used have been of Kuramoto [17, 18] or Kuramoto–Sakaguchi type [19–21], or Josephson junctions [22, 23]. We will build on these results, but to the best of our knowledge, this is the first application to theta neurons.

The structure of the paper is as follows. In Sect. 2 we consider instantaneous synaptic transmission between neurons, and in Sect. 3 we consider delayed synaptic transmission, where the delay is due to synaptic processing. Section 4 discusses four other related types of model neurons, whose networks can be analysed in a similar way to that in Sect. 2. We conclude in Sect. 5.

## Instantaneous Synapses

In this section we consider instantaneous synapses in the sense that the synaptic input to a neuron, in the form of a current, depends on the present state of the neurons which are connected to it.

### Derivation of Equations

Suppose we have a network of *N* ($3< N$) identical theta neurons, all-to-all coupled by instantaneous synapses. The dynamics is described by [24–27]

1 $$\begin{aligned} \frac{d\theta_{k}}{dt} =1-\cos{\theta_{k}}+(1+\cos{\theta_{k}}) (\eta+\kappa I ) \end{aligned}$$

for $k=1,2,\ldots, N$, where

2 $$ I=\frac{1}{N}\sum_{j=1}^{N}(1-\cos{ \theta_{j}})^{2}, $$

*κ* is the strength of coupling (which could be positive or negative), and *η* is the input current to all neurons when uncoupled. The *j*th term in the sum (2) represents the pulse of current emitted by the *j*th neuron as it fires, i.e. $\theta_{j}$ increases through *π*. The function $(1-\cos{\theta})^{2}$ is non-zero except when $\theta =0$, and thus this form of coupling may be regarded as non-physical, but the pulse can be localised more around $\theta=\pi$ by increasing the second power in (2) as will be discussed below. Note that each neuron is actually coupled to itself, but this term is only one out of *N* in the sum (2), so will be negligible for large *N*.

We can write (1) as

3 $$ \frac{d\theta_{k}}{dt}=\omega+\operatorname{Im} \bigl[\mathrm{He}^{-i\theta_{k}} \bigr], $$

where $\omega=\eta+\kappa I+1$ and $H=i(\eta+\kappa I-1)$. Note that *ω* is real and *H* is imaginary. The WS ansatz [9, 28] states that there is a transformation

4 $$ \tan{ \biggl(\frac{\theta_{k}(t)-\varPhi(t)}{2} \biggr)}=\frac{1-\rho (t)}{1+\rho(t)}\tan{ \biggl( \frac{\psi_{k}-\varPsi(t)}{2} \biggr)};\quad k=1,2,\ldots, N, $$

giving almost any solution of (1)–(2) ($\theta_{k}$) in terms of *N* constants ($\{\psi_{k}\}, k=1,2,\ldots, N$) and three variables ($\rho,\varPhi$ and *Ψ*), where these variables satisfy the ODEs

5 $$\begin{aligned} \frac{d\rho}{dt} & = \frac{1-\rho^{2}}{2}\operatorname{Re} \bigl[\mathrm{He}^{-i\varPhi} \bigr], \end{aligned}$$

6 $$\begin{aligned} \frac{d\varPhi}{dt} & = \omega+\frac{1+\rho^{2}}{2\rho}\operatorname {Im} \bigl[\mathrm{He}^{-i\varPhi} \bigr], \end{aligned}$$

7 $$\begin{aligned} \frac{d\varPsi}{dt} & = \frac{1-\rho^{2}}{2\rho}\operatorname{Im} \bigl[\mathrm{He}^{-i\varPhi } \bigr]. \end{aligned}$$

Thus, while it may seem possible that (for a given initial condition) a solution of (1)–(2) can explore the full *N*-dimensional phase space described by the *N* values of $\theta_{k}$, the solution is actually constrained to lie on a three-dimensional manifold with coordinates $\rho,\varPhi$ and *Ψ*. The dynamics on this manifold will depend on the values of the constants $\{\psi_{k}\}$.

There are *N* variables $\{\theta_{k}\}$, *N* constants $\{\psi_{k}\}$ and three variables $\rho,\varPhi$ and *Ψ*, and thus one needs to specify three constraints so that there is a unique relationship between $\{\theta_{i}\}$ and $\{\psi_{k},\rho,\varPhi,\varPsi\}$, to determine initial conditions, for example. One way to do this is to set $\rho(0)=\varPhi(0)=\varPsi(0)=0$, so that $\{\psi_{k}\}=\theta _{k}(0)$. Then integrate (5)–(7) with $\rho(0)=\varPhi (0)=\varPsi(0)=0$ and use the solutions $\rho(t),\varPhi(t)$ and $\varPsi(t)$ to reconstruct $\{\theta_{k}(t)\}$ using (4). Here, the constraints are on $\rho(0),\varPhi(0)$ and $\varPsi(0)$. Another set of constraints often taken is [28]

8 $$ \sum_{k=1}^{N} e^{i\psi_{k}}=0;\qquad \operatorname{Re} \Biggl[\sum_{k=1}^{N} e^{2i\psi _{k}} \Biggr]=0. $$

Given the *N* initial values $\theta_{k}(0)$, one can usually uniquely determine the values of $\rho(0),\varPhi(0)$ and $\varPsi(0)$ and $\{\psi_{k}\}$ such that both (4) and (8) hold [9]. “Usually” refers to solutions for which fewer than half of the neurons have exactly the same state. This rules out full synchrony, which is often a state of interest; however, we can understand the fully-synchronous state by simply considering the behaviour of one self-coupled neuron. We will use (8) unless specified otherwise.

In order to use (5)–(7), we need to write *I* (and thus *ω* and *H*) in terms of the new variables and the constants $\{\psi_{k}\}$. Now [28]

9 $$\begin{aligned} I & =3/2-\frac{1}{N}\sum_{j=1}^{N} \bigl(e^{i\theta_{j}}+e^{-i\theta _{j}} \bigr)+\frac{1}{4N}\sum _{j=1}^{N} \bigl[ \bigl(e^{i\theta _{j}} \bigr)^{2}+ \bigl(e^{-i\theta_{j}} \bigr)^{2} \bigr] \end{aligned}$$

10 $$\begin{aligned} & = 3/2-(z\gamma+\bar{z}\bar{\gamma})+ \bigl(z^{2} \gamma_{2}+ \bar{z}^{2}\bar {\gamma_{2}} \bigr)/4, \end{aligned}$$

where overbar indicates complex conjugate, $z=\rho e^{i\varPhi}$,

11 $$ \gamma=\frac{1}{N}\sum_{k=1}^{N} \frac{1+ \vert z \vert ^{-2}\bar{z}e^{i(\psi_{k}+\varPhi-\varPsi)}}{1+\bar{z}e^{i(\psi_{k}+\varPhi -\varPsi)}} =\frac{1}{N\rho}\sum_{k=1}^{N} \frac{\rho+e^{i(\psi_{k}-\varPsi )}}{1+\rho e^{i(\psi_{k}-\varPsi)}} $$

and

12 $$ \gamma_{2}=\frac{1}{N}\sum_{k=1}^{N} \biggl(\frac{1+ \vert z \vert ^{-2}\bar{z}e^{i(\psi_{k}+\varPhi-\varPsi)}}{1+\bar{z}e^{i(\psi _{k}+\varPhi-\varPsi)}} \biggr)^{2} =\frac{1}{N\rho^{2}}\sum _{k=1}^{N} \biggl(\frac{\rho+e^{i(\psi_{k}-\varPsi )}}{1+\rho e^{i(\psi_{k}-\varPsi)}} \biggr)^{2}. $$

We define

13 $$ C_{n}=\frac{1}{N}\sum_{k=1}^{N} e^{in\psi_{k}} $$

and see that $C_{0}=1$, and from (8), $C_{1}=0$. Using a series expansion of $[1+\rho e^{i(\psi_{k}-\varPsi)}]^{-1}$, we can write in the general case

14 $$ \gamma=1+ \bigl(1-1/\rho^{2} \bigr)\sum_{n=2}^{\infty}C_{n} \bigl(-\rho e^{-i\varPsi} \bigr)^{n}. $$

For the special case of $\psi_{k}=2\pi k/N$, i.e. evenly spaced $\psi_{k}$, $C_{n}=0$ except when *n* is a multiple of *N*, when it equals 1. Then (14) is a geometric series and

15 $$ \gamma=1+\frac{(1-1/\rho^{2})(-\rho e^{-i\varPsi})^{N}}{1-(-\rho e^{-i\varPsi})^{N}} $$

(note that this expression is given incorrectly in [17, 21]).

In the general case,

16 $$ \gamma_{2}=\sum_{n=0}^{\infty}(n+1) \bigl(-\rho e^{-i\varPsi} \bigr)^{n} \biggl[C_{n}+ \frac{2e^{-i\varPsi}}{\rho}C_{n+1}+\frac{e^{-2i\varPsi }}{\rho^{2}}C_{n+2} \biggr]. $$

For evenly-spaced $\psi_{k}$,

17 $$\begin{aligned} \gamma_{2}= {}& \sum_{k=0}^{\infty}(Nk+1) \bigl(-\rho e^{-i\varPsi} \bigr)^{Nk} +\sum _{k=1}^{\infty}Nk\frac{2e^{-i\varPsi}}{\rho} \bigl(-\rho e^{-i\varPsi } \bigr)^{Nk-1} \\ &{} +\sum_{k=1}^{\infty}(Nk-1)\frac{e^{-2i\varPsi}}{\rho^{2}} \bigl(-\rho e^{-i\varPsi} \bigr)^{Nk-2} \end{aligned}$$

18 $$\begin{aligned} ={}& N\sum_{k=1}^{\infty}k \bigl(-\rho e^{-i\varPsi} \bigr)^{Nk}+\sum_{k=0}^{\infty}\bigl(-\rho e^{-i\varPsi} \bigr)^{Nk} -\frac{2N}{\rho^{2}}\sum _{k=1}^{\infty}k \bigl(-\rho e^{-i\varPsi} \bigr)^{Nk} \\ &{} + \frac{N}{\rho^{4}}\sum_{k=1}^{\infty}k \bigl(-\rho e^{-i\varPsi } \bigr)^{Nk}-\frac{1}{\rho^{4}}\sum _{k=1}^{\infty}\bigl(-\rho e^{-i\varPsi} \bigr)^{Nk} \end{aligned}$$

19 $$\begin{aligned} = {}& 1+\frac{(1-1/\rho^{4})(-\rho e^{-i\varPsi})^{N}}{1-(-\rho e^{-i\varPsi })^{N}}+N\frac{(1-1/\rho^{2})^{2}(-\rho e^{-i\varPsi})^{N}}{[1-(-\rho e^{-i\varPsi })^{N}]^{2}}. \end{aligned}$$

Irrespective of $\psi_{k}$, if $\rho=1$ then $\gamma=\gamma_{2}=1$. If $\rho<1$ and $\{\psi_{k}\}$ are uniformly distributed, then as $N\rightarrow\infty$ we see that $\gamma\rightarrow1$ and $\gamma_{2}\rightarrow1$. In these cases ($\gamma=\gamma_{2}=1$) *I* becomes independent of *Ψ* and (5) and (6) decouple from (7), i.e. the dynamics becomes two-dimensional.

An alternative description of the dynamics of (1)–(2) when $N=\infty$ is given by writing the continuity equation governing the evolution of the probability density of *θ*s, $p(\theta,t)$ [26]. One can then use the Ott/Antonsen (OA) ansatz [29, 30] to reduce the dynamics of this evolution equation to a single complex equation for the evolution of the order parameter $z\equiv\int_{0}^{2\pi}p(\theta,t)e^{i\theta}\,d\theta$:

20 $$ \frac{dz}{dt}=i(\eta+kI) (1+z)^{2}/2-i(1-z)^{2}/2=i \omega z+H/2-\bar {H}z^{2}/2, $$

where *ω* and *H* are as above and $I=3/2-(z+\bar{z})+(z^{2}+\bar{z}^{2})/4$. Substituting $z=\rho e^{i\varPhi}$ into (20) and taking real and imaginary parts, we recover (5) and (6). Thus the OA ansatz corresponds to a special case of the WS ansatz: when $N=\infty$ and the constants $\{\psi_{k}\}$ are uniformly spread over $[0,2\pi ]$ [28]. Note that while the OA ansatz gives dynamics on an invariant manifold in the space of all $p(\theta,t)$, if the neurons are identical, the manifold is not attracting, and thus the full dynamics is not given by (20) and must be described using the WS ansatz [17, 28].

### Infinite *N*, Equally-Spaced Constants

We now consider the case of $N=\infty$ with uniform density of $\psi_{k}$. Thus we are interested in solutions of (20), written as

21 $$ \frac{dz}{dt}=i(\eta+\kappa I+1)z+i(\eta+\kappa I-1) \bigl(1+z^{2} \bigr)/2, $$

where

22 $$ I=3/2-(z+\bar{z})+ \bigl(z^{2}+\bar{z}^{2} \bigr)/4. $$

Note that (21)–(22) are invariant under $(z,t)\mapsto(\bar{z},-t)$, i.e. simultaneous reflection in the real axis and reversal of time. This has a significant effect on the possible dynamics.

#### Excitatory Coupling

First consider $\kappa=1$. From setting $d\rho/dt=0$ in (5) and recalling that *H* is imaginary, we find two sorts of fixed points of (21)–(22): (i) those with $\rho=1$ and (ii) those with $\varPhi=0$. From (6), those with $\rho=1$ satisfy

23 $$ 0=\omega+\operatorname{Im} \bigl[\mathrm{He}^{-i\varPhi} \bigr]=\eta+\kappa I+1+(\eta+\kappa I-1) \cos{\varPhi}, $$

where

24 $$ I=3/2-2\cos{\varPhi}+\cos{(2\varPhi)}/2. $$

**Fig. 1:**
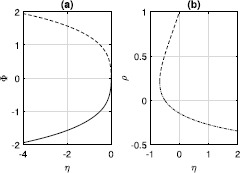
(**a**): Fixed points of (21)–(22) with $\rho=1$. Solid: stable, dashed: unstable. (**b**): Fixed points of (21)–(22) with $\varPhi=0$. Dash-dotted: focus, dashed: saddle. $\kappa=1$

These solutions are plotted in Fig. 1(a). Note that these solutions can be found directly from (1)–(2). For identical neurons, $\rho=1$ corresponds to full locking, so all $\theta_{i}$ are equal to *Φ* and a simple trigonometric identity gives (24) from (2).

Solutions of type (ii) with $\varPhi=0$ have $z=\rho$. From (6), they satisfy

25 $$ 0=\omega+\frac{1+\rho^{2}}{2\rho}\operatorname{Im}[H]=\eta+\kappa I+1+\frac {1+\rho^{2}}{2\rho}(\eta+ \kappa I-1), $$

where $I=3/2-2\rho+\rho^{2}/2$. These solutions (restricted to $-1\leq \rho\leq1$ for physical reasons) are shown in Fig. 1(b). Two solutions are created in a saddle-node bifurcation as *η* is increased (at a negative value of *η*). One is a saddle whose eigenvalues sum to zero, and the other is a focus with purely imaginary eigenvalues; these properties result from the reversibility of the system [31]. These fixed points correspond to *splay states* in which all neurons follow the same trajectory but are equally displaced from one another in time such that average quantities such as *z* are constant [9, 32, 33]. Note that the solutions shown in Fig. 1(a) collide with the saddle solution in Fig. 1(b) at $(\eta ,z)=(0,1)$.

**Fig. 2:**
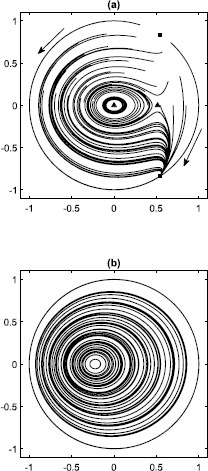
(**a**) Solutions of (21)–(22) in the *z* plane, with arrows showing the direction of increasing time. Fixed points are shown with solid squares ($\rho=1$) and triangles ($\varPhi=0$). $\eta=-0.5,\kappa=1$. (**b**) Solutions for $\eta=0.5,\kappa=1$

A selection of solutions and the fixed points are shown in Fig. 2(a) for $\eta=-0.5$. We see that for these parameter values, initial conditions either tend to the stable fixed point in the lower half plane (i.e. quiescence), or (if they are in the region enclosed by the homoclinic orbit to the saddle fixed point) follow one of a continuous family of periodic orbits. For $\eta>0$, the only remaining fixed point is the focus, and the phase space ($\rho\leq1$) is filled with a continuous family of periodic orbits (see Fig. 2(b)), again, as a result of the system’s reversibility.

#### Inhibitory Coupling

**Fig. 3:**
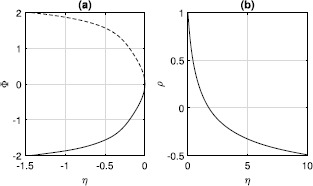
(**a**): Fixed points of (21)–(22) with $\rho=1$. Solid: stable, dashed: unstable. (**b**): Focus fixed point of (21)–(22) with $\varPhi=0$. $\kappa=-0.5$

Now consider $\kappa=-0.5$. As with excitatory coupling, there are fixed points with $\rho=1$, given by (23)–(24) and shown in Fig. 3(a). For $\eta>0$, there is also a focus fixed point (a splay state), as shown in Fig. 3(b). This fixed point is surrounded by a continuum of periodic orbits, as in Fig. 2(b). (Note that for strong inhibition, i.e. *κ* large and negative, the situation shown in Fig. 3(a) can become more complex, with multiple stable fixed points. This is due to the finite width of the pulses in (2), and the region of multistability disappears as the pulses are made narrower.)

In summary, the dynamics of (21)–(22) is non-generic due to their reversible nature, which can lead to the existence of continuous families of neutrally-stable periodic orbits.

### Finite *N*, Equally-Spaced Constants

We now consider the case of finite *N* but with equally-spaced $\{\psi _{k}\}$. Thus we consider (5)–(7) where *I* depends on *Ψ* via *γ* and $\gamma_{2}$. First we point out the reversibility of this system under the transformation $(\rho,\varPhi,\varPsi,t)\mapsto(\rho,-\varPhi,-\varPsi,-t)$. This transformation interchanges the products *zγ* and *z̄γ̄*, and $z^{2}\gamma_{2}$ and its conjugate. This leaves *I* (and thus *ω* and *H*) unchanged. Keeping in mind that *H* is imaginary, we see that this transformation leaves (5)–(7) unchanged, i.e. they are reversible.

#### Fixed Points

**Fig. 4:**
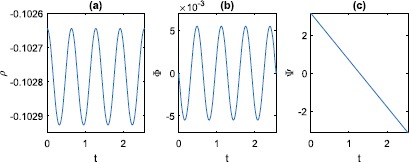
The periodic orbit corresponding to the focus fixed point in Fig. 1(b). $\kappa=1,\eta=-0.2,N=4$

The fixed points of type (i) with $\rho=1$ persist, independent of *N*, since these solutions have $\gamma=\gamma_{2}=1$. The values of *Φ* are given by solving (23)–(24). However, these fixed points have arbitrary values of *Ψ* since $d\varPsi/dt=0$. Regarding the fixed points of type (ii) that exist for $N=\infty$ as analysed in Sect. 2.2.1, they had constant and generically non-zero $d\varPsi/dt$. Thus, for finite *N*, we expect these to appear as time-dependent orbits, with the amplitudes of fluctuations in *ρ* and *Φ* going to zero as $N\rightarrow\infty$. To understand this, assume to a first approximation that *ρ* is constant. Then *γ* and $\gamma_{2}$ will have *N* periods of oscillation as *Ψ* goes through one period of oscillation. Thus $I,\omega$ and *H* will all have *N* oscillations in one period of *Ψ* and so will *ρ* and *Φ*. Thus the fixed points of type (ii) which exist for infinite *N* will appear for finite *N* as quasiperiodic orbits in which *ρ* and *Φ* undergo *N* oscillations for each full rotation in *Ψ*. An example for $N=4$ is shown in Fig. 4, where *Ψ* decreases from *π* to −*π*.

**Fig. 5:**
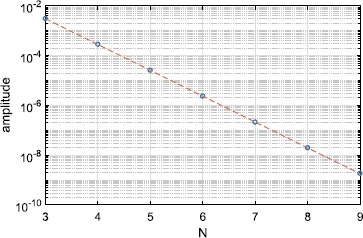
$\max(\rho)-\min(\rho)$ over one period for the type of periodic orbit shown in Fig. 4, as a function of *N* (circles). The dashed straight line is the best fit to the data. $\kappa=1,\eta=-0.2$

**Fig. 6:**
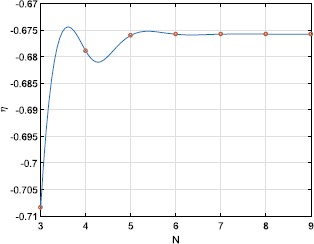
Location of the saddle-node bifurcation shown in Fig. 1(b) as a function of *N* (solid curve). The circles show interpolated values at integer *N*. $\kappa=1$

The amplitude of this type of periodic orbit goes to zero exponentially as $N\rightarrow\infty$; see Fig. 5. Even though *N* is physically an integer, the expressions for *γ* and $\gamma_{2}$ do not require it to be so. Thus we can, for example, continue the saddle-node bifurcation seen in Fig. 1(b) as a function of the continuous parameter *N*. The result is shown in Fig. 6, where we also show interpolated values at integer *N*. Interestingly, while the curve oscillates, the values at integer *N* are monotonic.

In terms of the original system (1)–(2), the periodic orbit shown in Fig. 4 corresponds to a periodic orbit with all Floquet multipliers having magnitude 1, i.e. completely neutrally stable. Two of the multipliers are a complex conjugate pair corresponding to the rotation seen in Fig. 2, while the remaining $N-2$ are equal to 1.

#### Other Orbits

**Fig. 7:**
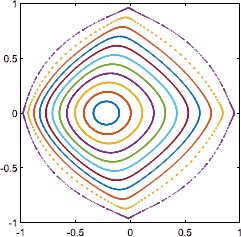
Values of *z* on the Poincaré section $\alpha\ (\mbox{mod } 2\pi)=0$, $d\alpha/dt>0$. 11 different initial conditions were used, and each orbit is shown with a different colour. $\kappa=1,\eta=0.5,N=4$

Consider the continuous family of periodic orbits which exist in the infinite-*N* case for $\kappa=1$ and *η* sufficiently positive (see Fig. 2(b)). These persist as what seems to be a continuous family of quasiperiodic orbits. Some are shown in Fig. 7 where we plot the value of *z* when $\alpha\equiv\varPhi-\varPsi$ increases through a multiple of 2*π*.

**Fig. 8:**
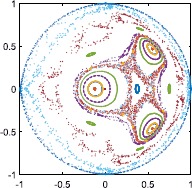
Values of *z* on the Poincaré section $\alpha\ (\mbox{mod } 2\pi)=0$, $d\alpha/dt>0$. Transients of duration 4000 were discarded. 15 different initial conditions were used, and each orbit is shown with a different colour. $\kappa=-0.5,N=4,\eta=0.6$

Now consider the case $\kappa=-0.5$. The dynamics in this case seems much more complex. An example is shown in Fig. 8 for $\eta=0.6$. For some initial conditions, the dynamics seems quasiperiodic, while for others the orbits appear to be chaotic (as indicated by a positive Lyapunov exponent, not shown). This mixture of quasiperiodic and chaotic behaviour has been previously observed in reversible systems [34] including a resistively-loaded series array of Josephson junctions also studied using the WS ansatz [22]. The overall trend for this system is that the dynamics becomes more regular as *η* is increased. We leave the investigation of this dynamics for a future publication.

### Finite *N*, Non-Uniform $\psi_{k}$

**Fig. 9:**
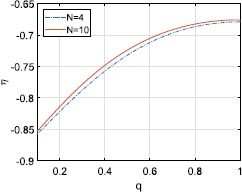
Location of the saddle-node bifurcation shown in Fig. 1(b) as a function of *q* for $N=4,10$. $\kappa=1$

**Fig. 10:**
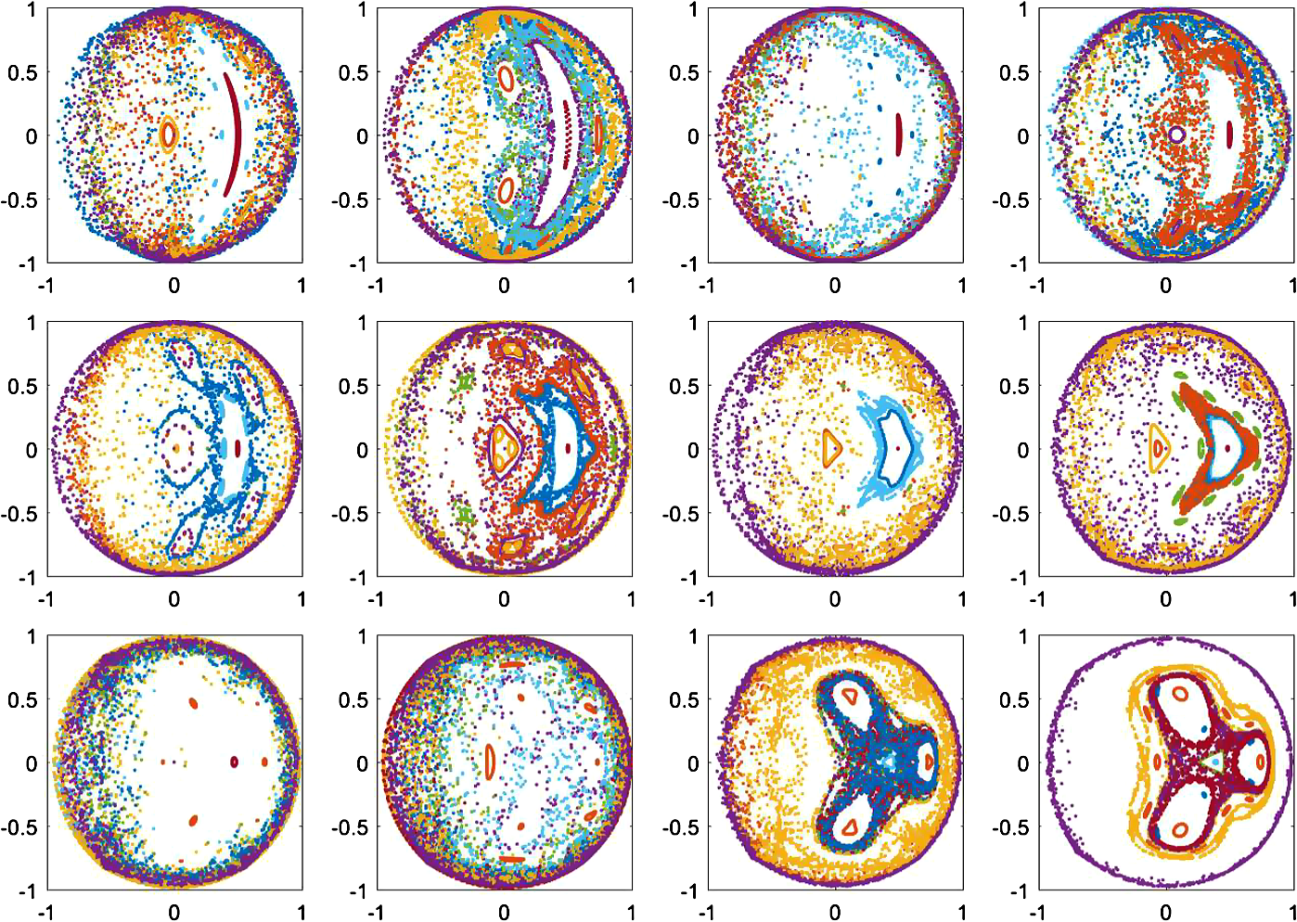
Values of *z* on the Poincaré section $\alpha\ (\mbox{mod } 2\pi)=0$, $d\alpha/dt>0$. *q* increases left to right, then top to bottom, in steps of $1/12$ starting at $q=1/12$ (top left). For each plot, 11 different initial conditions were used, and each orbit is shown with a different colour. $\kappa=-0.5,\eta=0.6,N=4$

Now consider non-uniformly spaced constants $\psi_{k}$. We follow [28] and distribute these values along two arcs each of length *qπ*, where $0< q\leq1$ is a parameter. Choosing *N* to be even, we have $\psi_{k}=(1-q)\pi/2-q\pi/N+2\pi qk/N$ for $k=1,2,\ldots, N/2$ and $\psi_{k}=(3-q)\pi/2-q\pi/N+2\pi qk/N$ for $k=N/2+1,2,\ldots, N$. For $q=1$, this is a uniform distribution, and as $q\rightarrow0$ the distribution tends to the two points $\pm\pi/2$. The saddle-node bifurcation shown in Fig. 1(b) moves as a function *q*, as shown in Fig. 9. Varying *q* for $\kappa<0$ also gives a variety of different dynamics, as shown in Fig. 10. Here we see a mixture of quasiperiodic and more complex behaviour, as seen in Fig. 8.

### Summary

In summary, for instantaneous synapses of the form studied here, there may be continuous families of periodic orbits (for drive $\eta>0$ and for some $\eta<0$ if $\kappa>0$) in the infinite-*N* uniformly distributed $\psi_{k}$ case. This is due to the reversibility of (21)–(22). For finite *N*, some of these orbits become quasiperiodic, with some initial conditions showing either quasiperiodic or more complex types of behaviour. This type of network can be thought of as having two sources of degeneracy: even if the constants $\{\psi_{k}\}$ are fixed, depending on parameters, there may be continuous families of neutrally stable periodic or quasiperiodic orbits. Choosing different initial conditions for the WS variables $\rho,\varPhi$ and *Ψ* selects between these orbits. Secondly, even for fixed initial conditions of the WS variables, there are many continuous families of orbits that can be obtained by varying $\{\psi_{k}\}$.

Note that if the system is bistable (for excitatory coupling and sufficiently small negative drive *η*), then even for fixed initial values of $\rho,\varPsi$ and *Φ*, changing either *N* (for evenly spaced constants $\psi_{k}$) or the distribution of $\psi_{k}$ (for fixed *N*) can lead to very different outcomes, as these changes can move the system from one basin of attraction to another.

## Synaptic Dynamics

We now consider including some synaptic processing, which amounts to delaying the synaptic input, still in the form of a current input. We thus replace (2) with

26 $$ \tau\frac{dI}{dt}=u-I, $$

where

27 $$ u=\frac{1}{N}\sum_{j=1}^{N}(1-\cos{ \theta_{j}})^{2}. $$

The network of neurons is still described by (5)–(7) but with the addition of

28 $$ \tau\frac{dI}{dt}=3/2-(z\gamma+\bar{z}\bar{\gamma})+ \bigl(z^{2} \gamma _{2}+\bar{z}^{2}\bar{\gamma_{2}} \bigr)/4-I, $$

where $z,\gamma$ and $\gamma_{2}$ are as in Sect. 2.1.

### Infinite *N*, Equally Spaced Constants

Repeating analysis as in Sect. 2.1, we now have

29 $$\begin{aligned} \frac{dz}{dt} & =i(\eta+\kappa I+1)z+i(\eta+\kappa I-1) \bigl(1+z^{2} \bigr)/2, \end{aligned}$$

30 $$\begin{aligned} \tau\frac{dI}{dt} & =3/2-(z+\bar{z})+ \bigl(z^{2}+ \bar{z}^{2} \bigr)/4-I. \end{aligned}$$

Fixed points of this pair of equations are the same as those of (21)–(22), but the stability of some of them has changed. Fixed points of type (i) shown in Fig. 1(a) gain another negative eigenvalue. The focus fixed point shown in Fig. 1(b) becomes stable, while the saddle fixed point in that figure remains a saddle, but with two stable directions. The continuum of periodic solutions shown in both panels of Fig. 2 is destroyed, and the system has either one stable fixed point, or two (in the region of bistability for $\eta<0$ but not too negative). In terms of attractors, (29)–(30) show what one would expect from an excitatorily self-coupled network. We have stable quiescence for large negative drive, bistability between quiescence and an active splay state for small negative drive, and a stable splay state for positive drive.

**Fig. 11:**
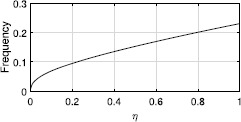
Frequency of the stable periodic orbit of (29)–(30). $\tau=1,\kappa=-0.5$

For the case of inhibitory coupling, fixed points of type (i) shown in Fig. 3(a) gain another negative eigenvalue (as for excitatory coupling). The focus fixed point in Fig. 3(b) now has one stable direction and two unstable ones. The continuum of periodic orbits seen for $\eta>0$ mentioned in Sect. 2.2.2 is now replaced by a single stable periodic orbit with $\rho=1$. The frequency of this orbit increases from zero as *η* does, as shown in Fig. 11.

### Finite *N*, Equally Spaced Constants

As was found in the case of instantaneous synapses, solutions with $\rho=1$ are unaffected by this change. However, for $\kappa=1$, the stable fixed point that exists for $\eta >0$ and $N=\infty$ now has small amplitude oscillations, as discussed in Sect. 2.3.1. The size of these oscillations decays exponentially with *N*, as was seen in Fig. 5. The only difference with the case discussed in Sect. 2.3.1 is that this low-amplitude periodic orbit is stable, whereas the one discussed in Sect. 2.3.1 was neutrally stable.

### Finite *N*, Unequally Spaced Constants

**Fig. 12:**
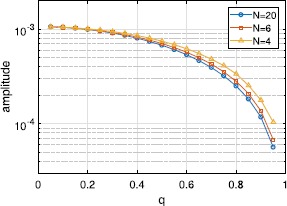
Amplitude (in *ρ*) of the stable periodic orbit of (29)–(30) as a function of *q* for $N=4,6,20$. $\tau=1,\kappa=1,\eta=1$

Here we distribute the constants $\psi_{k}$ as in Sect. 2.4 and investigate the effects of varying *q* on the amplitude of the oscillations in *ρ* of the stable periodic orbit that exists for $\kappa=1,\eta=1,\tau=1$. The results are shown in Fig. 12. For $q=1$, we saw previously that the amplitude of oscillations decays exponentially to zero as *N* increases. However, for $q<1$, the amplitude is always finite and has a limiting value as $N\rightarrow\infty$. As $q\rightarrow0$, the amplitude increases and the value of *N* becomes less relevant. This can be seen by examining the coefficients $C_{n}$ (13). For $q=0$, the first $N/2$ of $\psi_{k}$ equal $\pi/2$, while the last $N/2$ equal $-\pi/2$. Inserting this into (13), we find that $C_{n}=0$ for *n* odd, and $C_{2n}=(-1)^{n}$, independent of *N*.

### Summary

In summary, adding synaptic dynamics of the form examined in this section destroys the reversibility of (21) and thus the non-generic behaviour of solutions of that equation. In terms of attractors, (29)–(30) show what one would expect from a self-coupled network. The only effect of having finite *N* is to add small fluctuations to the stable splay state.

However, in the full network (1)–(2) there can still be continuous families of attracting periodic orbits near the splay state of (29)–(30) which differ in their initial conditions, and therefore their $\{\psi_{k}\}$. The way to understand this is to imagine picking $\{\theta_{i}(0)\}$. Setting $\rho=\varPsi=\varPhi=0$ gives $\{\psi_{k}\}$, via (4), which are now fixed. (These $\{\psi_{k}\}$ will typically not satisfy (8).) We can integrate (5)–(7) and (28) with these $\{\psi_{k}\}$ and essentially arbitrary $\rho(0),\varPsi (0),\varPhi(0)$ and $I(0)$, and since *κ* and *η* are both positive, this system will have only one attractor. On this attractor the dynamics will be determined by $\{\psi_{k}\}$. Hence different initial conditions of the $\{\theta_{i}(0)\}$ give different attractors. These orbits have $N-2$ Floquet multipliers of 1 and three which are less than 1 in magnitude. These three are associated with the stability of the splay state fixed point of (29)–(30).

## Other Similar Models

In this section we discuss several similar models and consider the case of identical oscillators.

### Gap Junction Coupling

Here we consider a network of all-to-all gap junctionally coupled theta neurons. Laing [25] showed, using the equivalence of a theta neuron and a quadratic integrate-and-fire neuron [35], that a network of *N* identical gap junctionally coupled theta neurons can be written as follows:

31 $$ \frac{d\theta_{j}}{dt}=1-\cos{\theta_{j}}-g\sin{\theta_{j}}+(1+ \cos {\theta_{j}}) (I+gQ), $$

where *g* is the strength of coupling, *I* is a constant input, and

32 $$ Q=\frac{1}{N}\sum_{k=1}^{N} \frac{\sin{\theta_{k}}}{1+\cos{\theta _{k}}+\epsilon}, $$

where $0<\epsilon\ll1$. We can write (31) as

33 $$ \frac{d\theta_{j}}{dt}=\omega+\operatorname{Im} \bigl[\mathrm{H e}^{-i\theta_{j}} \bigr], $$

where $\omega=1+I+gQ$ and $H=g+i(I+gQ-1)$. Thus the solutions of (31) can be described by the three ODEs (5)–(7) with the above definitions of *ω* and *H*. Extending the results of Laing [25], we find that

34 $$ Q=\sum_{m=1}^{\infty}b_{m} \gamma_{m} z^{m}+c.c., $$

where $z=\rho e^{i\varPhi}$, “c.c.” indicates the complex conjugate of the previous term,

35 $$ b_{m}=\frac{i(r^{m+1}-r^{m-1})}{2(r+1+\epsilon)} $$

$r\equiv\sqrt{2\epsilon+\epsilon^{2}}-1-\epsilon$ and

36 $$ \gamma_{m}\equiv\frac{1}{N}\sum_{k=1}^{N} \biggl(\frac{1+ \vert z \vert ^{-2}\bar{z}e^{i(\psi_{k}+\varPhi-\varPsi)}}{1+\bar {z}e^{i(\psi_{k}+\varPhi-\varPsi)}} \biggr)^{m}. $$

For $N=\infty$ and equally-spaced $\psi_{k}$, one can show [28] that all $\gamma_{m}=1$ and thus (5)–(6) are sufficient to describe this system. Written in complex form, (5)–(6) are

37 $$ \frac{dz}{dt}=i(1+I+gQ)z+ \bigl[g+i(I+gQ-1) \bigr]/2- \bigl[g-i(I+gQ-1) \bigr]z^{2}/2. $$

**Fig. 13:**
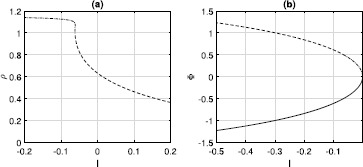
(**a**): Fixed point of (37) with $\rho\neq 1$. (**b**): Fixed points of (37) with $\rho=1$. Solid: sink; dashed: source; dash-dotted: saddle. $g=0.5,\epsilon=0.01$. The sum in (34) is truncated after 100 terms

Note that unlike (21)–(22), equation (37) with (34) is not invariant under $(z,t)\mapsto(\bar{z},-t)$ if $g>0$. In a similar way to the synaptically coupled network, (37) has two fixed points with $\rho=1$ for $I<0$ (these correspond to all neuron states being equal). From (5)–(6), these satisfy

38 $$ 0=I+gQ+1+(I+gQ-1)\cos{\varPhi}-g\sin{\varPhi}, $$

which could also be derived directly from (31). These fixed points are shown in Fig. 13(b).

For large and negative *I*, the fixed points with $\rho=1$ are a source and a sink, and there exists another unphysical saddle fixed point with $\rho>1$ (Fig. 13(a)). As *I* is increased, the unphysical solution is involved in a transcritical bifurcation with the source fixed point having $\rho=1$, and it enters the unit circle. As *I* is increased through zero, the synchronous fixed points are destroyed in a saddle-node on invariant circle (SNIC) bifurcation, leading to synchronous oscillations with $\rho=1$, with a source fixed point inside the unit circle.

The only attractors for this system have $\rho=1$, and for these it is clear that all $\gamma_{m}=1$ for finite *N* and any $\psi_{k}$, and thus their dynamics will still be described by (37) in this case. Solutions with $\rho<1$ (none of which are attracting) will, however, be described by (5)–(7), and their dynamics will depend on *N* and the distribution of $\psi_{k}$.

### Conductance Dynamics

Coombes and Byrne [36] considered a model in which synaptic input was in the form of a current, equal to the product of a conductance and the difference between the voltage of a quadratic integrate-and-fire neuron and a reversal potential. A particular case of their model can be written as follows:

39 $$ \frac{d\theta_{j}}{dt}=1-\cos{\theta_{j}}+(1+\cos{\theta_{j}}) \bigl[\eta +g(t)V_{\mathrm{syn}} \bigr]-g(t)\sin{\theta_{j}}, $$

where *η* is a constant drive, $V_{\mathrm{syn}}$ is the reversal potential (positive for excitatory coupling, negative for inhibitory one), and

40 $$ g(t)=\frac{2k}{N}\sum_{j=1}^{N}\delta \bigl(\theta_{j}(t)-\pi \bigr), $$

where *k* is the coupling strength ($0< k$) and *δ* is the Dirac delta. Equation (39) can be written as follows:

41 $$ \frac{d\theta_{j}}{dt}=\omega+\operatorname{Im} \bigl[\mathrm{H e}^{-i\theta_{j}} \bigr], $$

where $\omega=1+\eta+gV_{\mathrm{syn}}$ and $H=g+i(\eta+gV_{\mathrm{syn}}-1)$. Thus, as in Sect. 4.1, the solutions of (39) can be described by the three ODEs (5)–(7) with these definitions of *ω* and *H*. We have

42 $$ g=\kappa \Biggl[1+\sum_{m=1}^{\infty}(-z)^{m} \gamma_{m}+c.c. \Biggr], $$

where $z=\rho e^{i\varPhi}$, $\kappa=k/\pi$, and $\gamma_{m}$ is given in (36). For infinite *N* and equally spaced $\psi_{k}$, the system is described by

43 $$\begin{aligned} \frac{dz}{dt}={}&i(1+\eta+gV_{\mathrm{syn}})z+ \bigl[g+i(\eta+gV_{\mathrm{syn}}-1) \bigr]/2 \\ &{}- \bigl[g-i(\eta +gV_{\mathrm{syn}}-1) \bigr]z^{2}/2, \end{aligned}$$

where all $\gamma_{m}=1$. Synchronous fixed points have $\rho=1$ and $g=0$, and thus satisfy $0=1+\eta+(\eta-1)\cos{\varPhi}$, which we could obtain directly from (39). These fixed points only exist for $\eta\leq0$.

For excitatory coupling ($V_{\mathrm{syn}}=2,\kappa=1$), the fixed points are qualitatively the same as in Fig. 1, although the pair with $\rho\neq 0$ do not have $\varPhi=0$. This pair (analogous to those in panel (b) of Fig. 1) are a stable focus and a saddle, just as in Sect. 3.1, leading to a region of bistability. For inhibitory coupling ($V_{\mathrm{syn}}=-2,\kappa=1$), the fixed points are qualitatively the same as in Fig. 3; although, again, the fixed point for $\eta>0$ does not have $\varPhi=0$, and it is a stable focus.

We note that this model does not have the reversibility of that in Sect. 2, and thus none of the non-generic behaviour seen there. The analysis of this model for finite *N* should be similar to that in Sect. 3, but we do not present the results here.

### Winfree Model

The Winfree model of *N* pulse-coupled oscillators dates from 1967 [37–39] and is written for identical oscillators as

44 $$ \frac{d\theta_{i}}{dt}=\varOmega+\varepsilon\frac{Q(\theta_{i})}{N}\sum _{j=1}^{N} P(\theta_{j}), $$

where we choose $Q(\theta)=\sin{\beta}-\sin{(\theta+\beta)}$ and $P(\theta)=a_{n}(1+\cos{\theta})^{n}$ where $a_{n}$ is a constant such that $\int_{0}^{2\pi}P(\theta)\,d\theta=2\pi$. The function *Q* is the phase response curve of an oscillator, which can be measured experimentally or determined from a model neuron [40], and $P(\theta)$ is the pulsatile signal sent by a neuron whose state is *θ*. We can write (44) as

45 $$ \frac{d\theta_{i}}{dt}=\omega+\operatorname{Im} \bigl[\mathrm{H e}^{-i\theta_{i}} \bigr], $$

where $\omega=\varOmega+\varepsilon\sigma h$ and $H=\varepsilon e^{-i\beta}h$ and

46 $$ h=\frac{1}{N}\sum_{j=1}^{N} P( \theta_{j}). $$

Thus the solutions of (44) can be described by the three ODEs (5)–(7) with the above definitions of *ω* and *H*. To be concrete, choose $n=2$, in which case

47 $$ h=1+2(z\gamma+\bar{z}\bar{\gamma})/3+ \bigl(z^{2}\gamma_{2}+ \bar{z}^{2}\bar {\gamma_{2}} \bigr)/6, $$

where $z=\rho e^{i\varPhi}$ and *γ* and $\gamma_{2}$ are as given in (11)–(12). As for the network of theta neurons, in the case of $N=\infty$ and equally-spaced $\psi_{k}$, $\gamma=\gamma_{2}=1$ and (7) decouples from (5)–(6), which are, for this system,

48 $$\begin{aligned} \frac{d\rho}{dt} & =\varepsilon h \biggl(\frac{1-\rho^{2}}{2} \biggr)\cos{(\varPhi+ \beta)}, \end{aligned}$$

49 $$\begin{aligned} \frac{d\varPhi}{dt} & = 1+\varepsilon h \biggl[\sin{\beta}- \biggl( \frac {1+\rho^{2}}{2\rho} \biggr)\sin{(\varPhi+\beta)} \biggr], \end{aligned}$$

where we have rescaled time so that $\varOmega=1$, and $h=1+(4\rho /3)\cos{\varPhi}+(\rho^{2}/3)\times \cos{(2\varPhi)}$. Equations (48)–(49) are equations (12a) and (12b) in [37] once identical oscillators are considered. Equations (48)–(49) have two types of fixed point: (i) one for which $\varPhi=\pi/2-\beta$, with *ρ* satisfying

50 $$ 0=1+\varepsilon \bigl[1+(4\rho/3)\sin{\beta}- \bigl(\rho^{2}/3 \bigr) \cos{(2\beta )} \bigr] \bigl[\sin{\beta}- \bigl(1+\rho^{2} \bigr)/(2 \rho) \bigr] $$

and (ii) up to two for which $\rho=1$ (locked states), satisfying

51 $$ 0=1+\varepsilon \bigl[1+(4/3)\cos{\varPhi}+(1/3)\cos{(2\varPhi)} \bigr] \bigl[\sin{ \beta }-\sin{(\varPhi+\beta)} \bigr]. $$

**Fig. 14:**
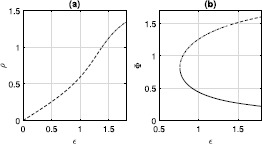
Fixed points of the system of Winfree oscillators, (48)–(49). (**a**): $\varPhi=\pi/2-\beta$. (**b**): $\rho=1$. Solid: sink; dashed: source; dash-dotted: saddle. Parameters: $\beta=0.1$

These fixed points and their stability are shown in Fig. 14 as a function of *ε* for $\beta=0.1$. For small *ε*, the only attractor is a periodic orbit with $\rho=1$ corresponding to synchronous oscillations. As *ε* is increased, there is a saddle-node on invariant circle (SNIC) bifurcation destroying the periodic orbit and leading to the creation of type (ii) fixed points, one of which is stable. As *ε* is increased further, the type (i) fixed point undergoes a transcritical bifurcation with the saddle fixed point on $\rho=1$ and leaves the unit circle, thereby becoming unphysical. This sequence of bifurcations happens for all $0\leq\beta<\pi/2$. Note the similarity between this scenario and that for the gap junction coupled neurons in Sect. 4.1: both have a SNIC bifurcation on the circle $\rho =1$, with the saddle later becoming a source as another fixed point leaves the unit circle.

As was the case in Sect. 4.1, since the only attractors have $\rho=1$, these will persist unchanged for finite *N* and any $\psi_{k}$, as *γ* and $\gamma_{2}$ will both equal 1 in this case.

### QIF Neurons

The correspondence between a theta neuron and a quadratic integrate-and-fire (QIF) neuron is well known [11, 25, 41]. Thus, some of the degenerate behaviour seen here in the networks of identical theta neurons should also appear in all-to-all coupled networks of identical QIF neurons [32]. Consider the network in [41]:

52 $$ \frac{dV_{i}}{dt}=V_{i}^{2}+I_{0}+Jr(t) $$

for $i=1,2,\ldots, N$, with the rule that when $V_{i}=\infty$ (i.e. neuron *i* fires) $V_{i}$ is set to −∞. Here, $I_{0}$ is a constant, *J* is the strength of coupling between the neurons, and $r(t)$ is the rate at which the network is firing:

53 $$ r(t)=\frac{1}{N}\sum_{j=1}^{N}\sum _{k}\delta \bigl(t-t_{j}^{k} \bigr), $$

where $t_{j}^{k}$ is the *k*th firing time of the *j*th neuron, and the sum over *k* is only over past firing times. Using the transformation $V_{j}=\tan{(\theta_{j}/2)}$, (52) becomes

54 $$ \frac{d\theta_{j}}{dt}=1-\cos{\theta_{j}}+(1+\cos{\theta_{j}}) (I_{0}+Jr), $$

which is the same form as (1). Thus, under the assumption of infinite *N* and equally spaced constants $\psi_{k}$, the dynamics of (54) is governed by

55 $$ \frac{dz}{dt}=i(I_{0}+Jr+1)z+i(I_{0}+Jr-1) \bigl(1+z^{2} \bigr)/2 $$

(see (21)). The authors [41] showed that defining

56 $$ w=\frac{1-\bar{z}}{1+\bar{z}} $$

equation (55) can be written

57 $$ \frac{dw}{dt}=i(I_{0}+Jr)-iw^{2} $$

and that the real part of *w*, divided by *π*, is the firing rate *r*, while the imaginary part of *w* is the mean of the voltages $V_{i}$ in the original network (52). Thus writing $w=\pi r+i\widehat{V}$, (57) can be written as two real equations:

58 $$\begin{aligned} \frac{dr}{dt} & = 2r\widehat{V}, \end{aligned}$$

59 $$\begin{aligned} \frac{d\widehat{V}}{dt} & = \widehat{V}^{2}+I_{0}+Jr- \pi^{2}r^{2}. \end{aligned}$$

Note the reversibility: $(\widehat{V},t)\mapsto(-\widehat{V},-t)$. Equations (58)–(59) are the same as equations (12a) and (12b) in [41], once identical neurons are considered. Equations (58)–(59) will describe the dynamics of (52) in the limit $N\rightarrow\infty$, and when the constants $\psi_{k}$ are equally spaced. We now briefly discuss initial conditions for (52) and their relationship to $\psi_{k}$. Using (4) and $V_{k}=\tan{(\theta_{k}/2)}$, we have

60 $$ V_{k}=\tan{ \biggl\{ \frac{\varPhi}{2}+\tan^{-1} \biggl[ \frac{1-\rho }{1+\rho}\tan{ \biggl(\frac{\psi_{k}-\varPsi}{2} \biggr)} \biggr] \biggr\} }. $$

Equally-spaced $\psi_{k}$ means $\psi_{k}=2\pi k/N$, $k=1,2,\ldots, N$, and since tan is periodic with period *π*, we see that in the limit $N\rightarrow \infty$, the value of *Ψ* becomes irrelevant in determining $V_{k}$. (We expect this, as in this case—as we have seen a number of times—(5)–(6) decouple from (7).) For finite *N*, we will set $\varPsi=0$ and initialise $V_{k}$ as

61 $$ V_{k}(0)=\tan{ \biggl\{ \frac{\varPhi}{2}+\tan^{-1} \biggl[ \frac{1-\rho }{1+\rho}\tan{ \biggl(\frac{\pi k}{N} \biggr)} \biggr] \biggr\} }. $$

Varying *Φ* and *ρ* corresponds to moving through a two-parameter family of initial conditions for (52) which, in the limit $N\rightarrow\infty$, corresponds to varying the initial conditions of (58)–(59).

**Fig. 15:**
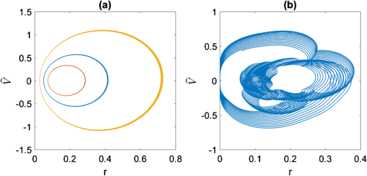
(**a**): Mean voltage *V̂* and firing rate *r*, extracted from simulations of the network (52). The three different curves correspond to three different choices of *Φ* and *ρ* in (61). (**b**): Results of a single simulation in which $V_{k}(0)$ were randomly chosen from a unit normal distribution. Simulations are of duration 100 time units. Parameters: $I_{0}=0.2, J=0.1,N=1000$

We implemented (52) just as in [41] but with $N=1000$ and identical neurons. Setting $I_{0}=0.2, J=0.1$ and using three different values of *Φ* and *ρ* to initialise $V_{k}$, we obtained the results in Fig. 15(a). (Both the mean voltage and firing rate were smoothed by convolving with a Gaussian of standard deviation 0.07 time units.) We see three out of a continuous family of periodic orbits. They match very closely numerical simulations of (58)–(59) (not shown) even though (58)–(59) are only valid in the limit $N\rightarrow\infty$. Conversely, if we choose $V_{k}(0)$ not using (61), for example, taking them randomly from a unit normal distribution, we obtain the results in Fig. 15(b). The solution is quasiperiodic and clearly not described by a planar system. To understand this, consider the transformation (4) with $\theta_{k}(0)=2\tan^{-1}(V_{k}(0))$. For large *N*, it is generally impossible to choose $\varPhi(0),\varPsi(0)$ and $\rho(0)$ such that $\psi_{k}$ are uniformly distributed. Thus the system must be described by three coupled equations of the form (5)–(7) which, even in the limit $N\rightarrow\infty$, will not reduce to a planar system due to the non-uniformity of the distribution of $\psi_{k}$.

Note that while (52) is synaptically driven by the instantaneous rate *r*, the network (1) is driven by the average of the pulse-like functions in (2). However, replacing $(1-\cos{\theta})^{2}$ in (2) by $a_{n}(1-\cos{\theta})^{n}$, where $a_{n}$ is chosen so that the integral of this function over $[0,2\pi]$ is independent of *n*, and taking the limit $n\rightarrow\infty$, one can obtain a drive equal (up to a scale factor) to the rate given in terms of delta functions in (53) [24, 36].

## Discussion and Conclusion

In conclusion, we studied finite and infinite networks of identical all-to-all coupled theta neurons, with both instantaneous and delayed synaptic interactions, and several related models (gap junction-coupled theta neurons, theta neurons with conductance dynamics, Winfree oscillators and quadratic integrate-and-fire neurons). For all models, the WS ansatz gives a reduced description of the model in terms of three ODEs and a set of constants. Changing these constants while keeping the initial conditions of the WS variables fixed gives different dynamics in the original models, and thus there is a continuum of such solutions. For instantaneous synapses, even keeping the constants fixed, there can be many solutions, due to either the reversibility of the dynamics of the WS variables, or the coexistence of many quasiperiodic orbits. It is only this case, of instantaneous synapses, which seems non-generic. Adding synaptic processing destroys this reversibility, and none of the other models (gap junction-coupled theta neurons, theta neurons with conductance dynamics or Winfree oscillators) have such a reversibility.

We have considered identical sinusoidally-coupled oscillators, for which the WS ansatz [9] gives the correct description in the form of the three ODEs (5)–(7), together with the constants $\{ \psi_{k}\}$. This is an idealisation, so we should discuss the case of non-identical oscillators. For an infinite number of non-identical oscillators, one can consider the continuity equation governing the evolution of the probability density of the phases [9, 26, 29]. Solutions of this decay onto the Ott/Antonsen (OA) manifold, on which their dynamics can be found using the OA ansatz [24–26, 29, 30, 37, 42, 43]. The OA equations are simpler than those resulting from the Watanabe/Strogatz ansatz: only a pair of real equations are needed to describe (1)–(2) when the value of *η* for each neuron is different, for example, rather than (5)–(7) and $\{\psi_{k}\}$ (although we must take $N\rightarrow\infty$ for them to be valid). The OA equations typically have hyperbolic and isolated fixed points, unlike those obtained using the Watanabe/Strogatz ansatz, which can be perturbed to a nearby similar state by varying one of $\psi_{k}$, for example. However, there remains a gap: is there a dimension reduction applicable to finite networks of non-identical sinusoidally-coupled oscillators? See [44] for ideas in this direction. Another approach is that of [28] who consider a number of subpopulations, each of which has identical oscillators although parameters are different between subpopulations, and the WS ansatz can be applied to each subpopulation.

There are several extensions that could be performed using the ideas presented here. The first is time-dependant forcing of the network [45]. As long as each neuron experiences the same force, the WS ansatz will apply. The second involves each neuron receiving common noise [46, 47]. Again, the WS ansatz will apply. We could also consider introducing a discrete delay in the synaptic processing, i.e. replacing $I(t)$ in (1) by $I(t-\tau)$ for some delay $\tau>0$. Another extension involves investigating a pair of populations of neurons, one excitatory and one inhibitory [41]. Coupling these may result in a PING rhythm [48], and if neurons are identical within each population, the coupled network may show interesting dynamics.

## Abbreviations

WS: Watanabe/Strogatz
SNIC: saddle-node on invariant circle
ODE: ordinary differential equation
QIF: quadratic integrate and fire
OA: Ott/Antonsen
